# Nanoparticles in Bone Regeneration: A Narrative Review of Current Advances and Future Directions in Tissue Engineering

**DOI:** 10.3390/jfb15090241

**Published:** 2024-08-23

**Authors:** Samira Farjaminejad, Rosana Farjaminejad, Franklin Garcia-Godoy

**Affiliations:** 1 School of Health and Psychological Sciences, Department of Health Services Research and Management, City University of London, London WC1E 7HU, UK; rosana.farjaminejad@city.ac.uk; 2 Department of Bioscience Research, Bioscience Research Center, College of Dentistry, University of Tennessee Health Science Center, 875 Union Avenue, Memphis, TN 38163, USA; fgarciagodoy@gmail.com

**Keywords:** bone tissue engineering, nanoparticle, bone regeneration, metal nanoparticles

## Abstract

The rising demand for effective bone regeneration has underscored the limitations of traditional methods like autografts and allografts, including donor site morbidity and insufficient biological signaling. This review examines nanoparticles (NPs) in tissue engineering (TE) to address these challenges, evaluating polymers, metals, ceramics, and composites for their potential to enhance osteogenesis and angiogenesis by mimicking the extracellular matrix (ECM) nanostructure. The methods involved synthesizing and characterizing nanoparticle-based scaffoldsand integrating hydroxyapatite (HAp) with polymers to enhance mechanical properties and osteogenic potential. The results showed that these NPs significantly promote cell growth, differentiation, and bone formation, with carbon-based NPs like graphene and carbon nanotubes showing promise. NPs offer versatile, biocompatible, and customizable scaffolds that enhance drug delivery and support bone repair. Despite promising results, challenges with cytotoxicity, biodistribution, and immune responses remain. Addressing these issues through surface modifications and biocompatible molecules can improve the biocompatibility and efficacy of nanomaterials. Future research should focus on long-term in vivo studies to assess the safety and efficacy of NP-based scaffolds and explore synergistic effects with other bioactive molecules or growth factors. This review underscores the transformative potential of NPs in advancing BTE and calls for further research to optimize these technologies for clinical applications.

## 1. Introduction

The demand for bone regeneration is rising because of bone diseases like infections, tumors, and bone loss [1]. This is crucial in orthopedics, dentistry, and reconstructive surgery, affecting millions globally [2]. Natural bone healing is complex and can be hindered by large defects, poor vascularization, and conditions like osteoporosis or diabetes [3]. Traditional methods include autografts, allografts, and synthetic substitutes [4]. Autografts are ideal due to their biocompatibility and osteoinductive potential but have limitations like donor site morbidity, limited supply, and infection risk [5]. Synthetic substitutes provide structural support but often lack the necessary biological signaling [5]. These challenges necessitate innovative approaches like TE, involving osteoprogenitor cell recruitment, differentiation, and bone matrix formation supported by porous biodegradable scaffolds [5,6,7]. Advances in scaffold materials aim to enhance osteogenesis and angiogenesis, mimicking bone’s natural architecture [8,9]. Despite progress, the exact mechanisms by which mechanical stimuli affect stem cell behavior are not fully understood [10]. Efforts to mimic the ECM’s nanostructure have led to scaffolds made from nanofibers, nanotubes, NPs, and hydrogels, which stimulate cell growth and guide tissue regeneration due to their biomimetic features [11,12]. NPs provide scaffolds for bone growth, deliver bioactive molecules, and modulate the immune response, offering minimally invasive, targeted therapy [13,14]. They have emerged as a groundbreaking tool in TE and regenerative medicine (TERM) due to their low toxicity, customizable characteristics, and targeted delivery potential [15,16]. These properties allow for the precise regulation of biological processes and real-time monitoring, enhancing the quality of engineered tissues and addressing challenges in TE and TERM [17]. NPs also allow for high control over scaffold properties and the controlled release of bioactive agents [18].

Additionally, the limitations of bioactive molecules—such as poor solubility, unstable bioactivity, and a short circulation half-life—can be mitigated using NPs, making them highly suitable for delivering and monitoring bioactive agents. Depending on the application, materials such as polymers, metals, ceramics, and their composites can be used to produce NPs, each offering unique advantages for TERM applications [19].

**Figure 1:**
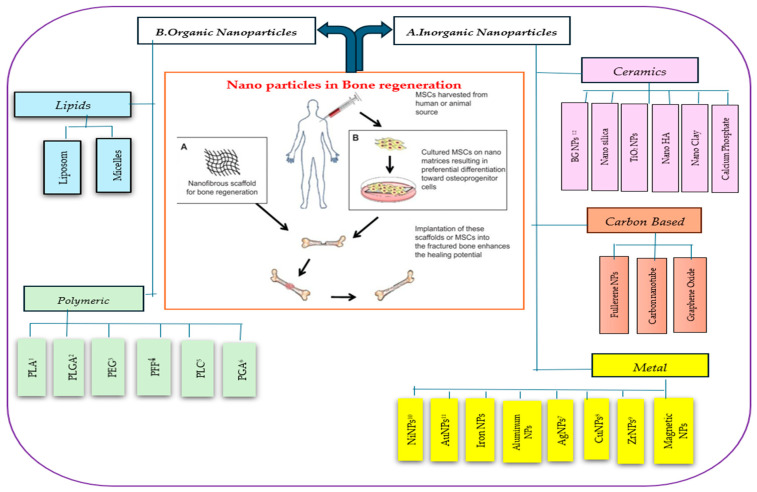
Classification of NPs in BTE. A. Inorganic NPs: includes metals (e.g., Ag, C, Zr, Ni, Au, Aluminum), and ceramics (e.g., HA). B. Organic NPs: includes lipid-based materials and polymers (e.g., chitosan), PLA, PLGA, PEG, PFF, PCL, and PGA, poly(glycolic acid). 1 PLA, poly(lactic acid). 2 PLGA, poly(lactic-glycolic acid). 3 PEG, polyethylene glycol. 4 PFF, poly(propylene fumarate). 5 PCL, polycaprolactone. 6 PGA, poly(glycolic acid). 7 Ag, silver. 8 Cu, Copper. 9 Zr, zirconium. 10 Ni, nickel. 11, Au, gold.

NPs can be prepared from a variety of materials, including ceramics, metals, and natural and synthetic polymers (Figure 1), each offering unique advantages like high penetration ability, high surface area with tunable surface properties [20]. These characteristics make NPs highly preferred in the TERM field for applications such as imaging, mechanical strength enhancement, bioink supplements, antimicrobial, and bioactive agent carriers [21]. This versatility underscores the critical role of NPs in advancing TERM technologies and applications [21].

Among these materials, polymers are particularly significant in developing biomaterials due to their versatility and adaptability at different scales (nano-, micro-, and macro-). They can be engineered to meet specific mechanical, chemical, and biological requirements, making them suitable for a wide range of biomedical applications. The adaptability of polymers allows them to be used in creating nano-scaled particles for drug delivery, micro-scaled scaffolds for TE, and macro-scaled implants for structural support in bone regeneration. This versatility underscores the critical role of polymers in advancing TERM technologies and applications [22].

**Figure 2:**
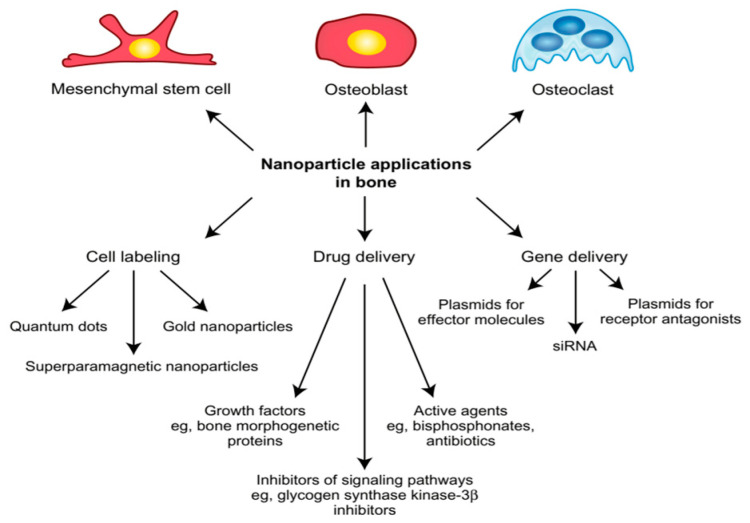
Summary of NP Applications in Bone, reprinted from Ref. [23].

Increasingly refined NPs are being developed for a wide range of applications (Figure 2). These include cell labeling to expand research possibilities and improve noninvasive monitoring of cell therapy approaches. Additionally, advanced drug delivery systems are being created to enhance pharmacological properties, enabling the controlled release of bioactive molecules like growth factors or anticancer drugs, thus improving therapeutic outcomes. Furthermore, promising gene therapy concepts are necessary for future treatments involving intracellular manipulation. Given the significant potential of NPs in these areas, this discussion focuses on their application in bone cells and tissue [23].

Nanomedicine enhances tissue regeneration by enabling controlled, sustained drug delivery through 3D porous scaffolds. Techniques like anodization, micelle lithography, and chemical vapor deposition create drug-loaded NPs. These NPs facilitate the release of growth factors, promoting tissue growth and repair. Specific NPs, such as iron oxide-gold and graphene oxide composites, have shown success in nerve growth and bone regeneration. NPs such as superparamagnetic microspheres, cerium oxide NPs, and gold NPs are used for cardiac tissue and wound healing. Integrating nanomedicine with TE aims to develop advanced scaffolds for regenerating various tissues, including nerves, bones, cardiac, and skeletal muscles. This integration results in smart, efficient scaffolds for effective tissue repair and regeneration [24].

This review aims to provide a comprehensive overview of the current advances in the use of NPs for bone regeneration and to discuss potential future directions in TE applications. The paper is structured into four main sections. The introduction highlights the increasing demand for effective bone regeneration and the limitations of traditional methods, emphasizing the potential of NPs to enhance osteogenesis and angiogenesis by mimicking the ECM nanostructure. It discusses various types of NPs, including polymers, metals, ceramics, and composites, and their integration with materials like HAp and polymers such as PLGA, PEG, and PPF, while also highlighting carbon-based NPs like graphene and carbon nanotubes. The results section analyzes the performance of these NPs in creating biocompatible and customizable scaffolds that enhance drug delivery and bone repair. The conclusion summarizes the key findings, discusses their implications for TE, and suggests future research directions to optimize nanoparticle technologies for clinical applications. The literature search strategy involved a comprehensive review of recent articles from key databases such as PubMed, Scopus, and Web of Science, focusing on studies published within the last decade. The inclusion criteria were based on relevance to the topic, novelty, and quality of research methodology, while studies that lacked sufficient experimental data or did not directly address the use of NPs in bone regeneration were excluded.

## 2. Inorganic NPs

Inorganic NPs, including ceramic NPs, carbon-based NPs, and metal NPs, are highly promising in bone regeneration due to their biocompatibility and biodegradability. Carbon-based NPs like fullerenes and carbon nanotubes are particularly noted for their biocompatibility, integrating well with bodily tissues. These properties enhance drug delivery, improve scaffold properties, and support cell proliferation and differentiation, making organic NPs vital in advancing TE for effective bone regeneration [25,26].

### 2.1. Ceramic NPs

#### 2.1.1. Nanohydroxyapatite (Nano-HA) for Bone Regeneration in TE

Nano-HA represents a significant advancement in bone regeneration technologies, providing superior interaction with biological tissues due to its nanoscale features that mimic the dimensions of natural bone components. This material is integral to enhancing osteoconductivity, osteoinductivity, and biocompatibility in TE applications [27].

Nano-HA is characterized by a high surface area-to-volume ratio, which improves its mechanical properties and bioactivity compared to its microscale counterparts. This makes nano-HA an excellent scaffold material that can support the attachment and proliferation of osteoblasts, thereby enhancing new bone formation [28]. The nanostructure of HA also facilitates the adsorption of proteins and growth factors, further promoting the osteoinduction process where new bone tissue is formed at the implant site [29].

Recent developments in nanotechnology have led to the creation of nano-HA particles through various synthesis methods such as wet chemical precipitation, sol–gel synthesis, and hydrothermal techniques. These methods allow for precise control over the particle size, crystallinity, and morphology of nano-HA, which are critical factors that affect the material’s performance in biological environments [28].

The application of nano-HA in TE involves its use as a coating for metallic implants to improve their integration with bone tissue, as well as its incorporation into composites and scaffolds that are used for bone defect repair [30,31]. Nano-HA has shown promising results in not only acting as a substitute for autologous bone grafts but also in enhancing the mechanical and biological performance of biomaterials used in orthopedics and dentistry [32].

Given its potential, nano-HA is being studied extensively for its role in next-generation bone regeneration strategies, where it is combined with other biomaterials to create hybrid systems that offer enhanced performance characteristics for clinical applications. As research progresses, the integration of nano-HA with other nanotechnologies is anticipated to lead to even more effective solutions for BTE, addressing a wide range of orthopedic and dental needs [28].

#### 2.1.2. Titanium Oxide Nanotubes in Bone Regeneration

Titanium and its alloys are widely used in total joint replacements. However, surface degradation from corrosion and wear releases debris, including ions and micro- and NPs, which contribute to particle-induced osteolysis and implant loosening. Effective cell-to-cell communication among various cell types is essential for bone healing and regeneration at the implant-bone interface [29]. Besides the internal cellular response to the uptake and intracellular localization of wear debris, there is concern that titanium dioxide nanoparticles (TiO_2_ NPs), which mimic wear debris, can disrupt cellular communication with surrounding tissue. This disruption may affect the balance between bone tissue integrity and regenerative processes [30].

Research indicates that TiO_2_ nanoparticles (NPs) promote the secretion of exosomes (Exos) from both immature and mature osteoblasts, each exhibiting unique proteomic profiles. Functional tests demonstrated that Exos originating from these osteoblasts reduce the osteogenic differentiation of human mesenchymal stem/stromal cells (HMSCs). Osteoblasts, derived from MSCs, coexist in the bone microenvironment during development and remodeling [29].

TiO_2_ nanotubes have emerged as a significant advancement in the field of bone regeneration due to their unique properties and interactions with bone cells. These nanotubes are fabricated through an electrochemical anodization process, which allows precise control over their nanoscale features. This control is crucial because the nanostructure of TiO_2_ can be tailored to enhance osseointegration, the process where bone cells bind directly to the material without any fibrous tissue in between [29].

The effectiveness of TiO_2_ nanotubes in bone regeneration can be attributed to their ability to mimic the natural ECM, a critical aspect of bone tissue that supports cell adhesion and growth. Studies have shown that the nanoscale dimensions and surface topology of these nanotubes promote the adhesion and proliferation of osteoblasts, the cells responsible for new bone formation. The enhanced surface area of the nanotubes increases protein adsorption, which is beneficial for cell attachment and spreading [30].

Moreover, the interaction of osteogenic cells with TiO_2_ nanotube surfaces has been found to stimulate osteogenic differentiation, particularly when the nanotubes are used in conjunction with osteoinductive biochemical cues. This synergy between the physical properties of the nanotubes and biological signals leads to improved bone tissue formation [30].

Additionally, the nanostructured surface of TiO_2_ allows for the modulation of mechanical properties. Interaction with MSCs is vital for bone healing and regeneration, as MSCs can differentiate into bone-forming cells under the right conditions [30,31].

Research has also explored the potential of TiO_2_ nanotubes as carriers for drugs and growth factors. The inner spaces of the nanotubes can be loaded with substances that promote bone growth or prevent infection, which are then released in a controlled manner to enhance the healing process [30,31,32].

TiO_2_ nanotubes represent a promising avenue in nanoparticle technology for bone regeneration. Their ability to enhance cell response, facilitate the delivery of biological agents, and integrate seamlessly with bone tissue underscores their potential in orthopedic applications. Moving forward, further studies and clinical trials are needed to fully realize and optimize the use of TiO_2_ nanotubes in medical implants and bone repair strategies [30,31,32].

#### 2.1.3. Nanosilica for Bone Regeneration in TE

Nanosilica, particularly mesoporous silica NPs, has shown significant potential in the domain of bone regeneration due to its unique physicochemical properties. These NPs are highly biocompatible, and their ordered mesoporous structure enables them to be excellent carriers for drug delivery, which is crucial for enhancing bone repair and regeneration [33,34].

A robust biosilicification strategy has been developed to create nanosilica-collagen (nSC) scaffolds derived from porcine demineralized cancellous bone (DCB). These scaffolds possess a porous structure that closely resembles native bone, with a uniform nanosilica coating. The osteoinductivity of these scaffolds is significantly influenced by the surface roughness and the silicon content of the silica coating. This innovative approach ensures uniform and complete internal and external surface coverage of a nanosilica coating throughout the scaffold, enhancing its mechanical properties and osteoinductive potential [4]. nSC scaffolds have demonstrated effectiveness in repairing critical-sized bone defects in animal models without the need for exogenous cells or growth factors. The scaffolds’ topographic and chemical cues activate multiple signaling pathways related to MSC recruitment and bone regeneration. This cell- and GF-free, one-step implantation approach shows high potential for clinical translation in treating large bone defects [33].

#### 2.1.4. Nanoclay

Nanoclays, a promising subset of the nanomaterial family, have garnered attention in TE, particularly for bone regeneration. Their unique properties, including high surface area, dual charge distribution, and excellent biocompatibility, make them ideal for reinforcing biomaterials. This reinforcement enhances both mechanical properties and bioactivity, which is crucial for effective BTE [35,36]. Montmorillonite (MMT), Halloysite Nanotubes (HNT), and Laponite RD are the three primary nanoclays used in bone regeneration. Each has distinct structural and compositional features that contribute to their effectiveness in biomedical applications [37]. MMT consists of layers of alumina octahedral sheets between two silica tetrahedral sheets, offering a high surface area and the ability to enhance cell proliferation and differentiation while suppressing osteoclast formation [36]. HNT, an aluminosilicate clay mineral, naturally forms nanotubes that provide high mechanical strength and biocompatibility, making them suitable for gene and drug delivery, TE, and bioimaging [38]. Laponite RD, a synthetic trioctahedral smectite, has a disk-shaped morphology and unique dual charge distribution and is extensively used in hydrogels and scaffolds due to its excellent exfoliation capacity and ability to enhance mechanical properties and support bioactivity necessary for tissue formation [36,37,38,39].

**Figure 3:**
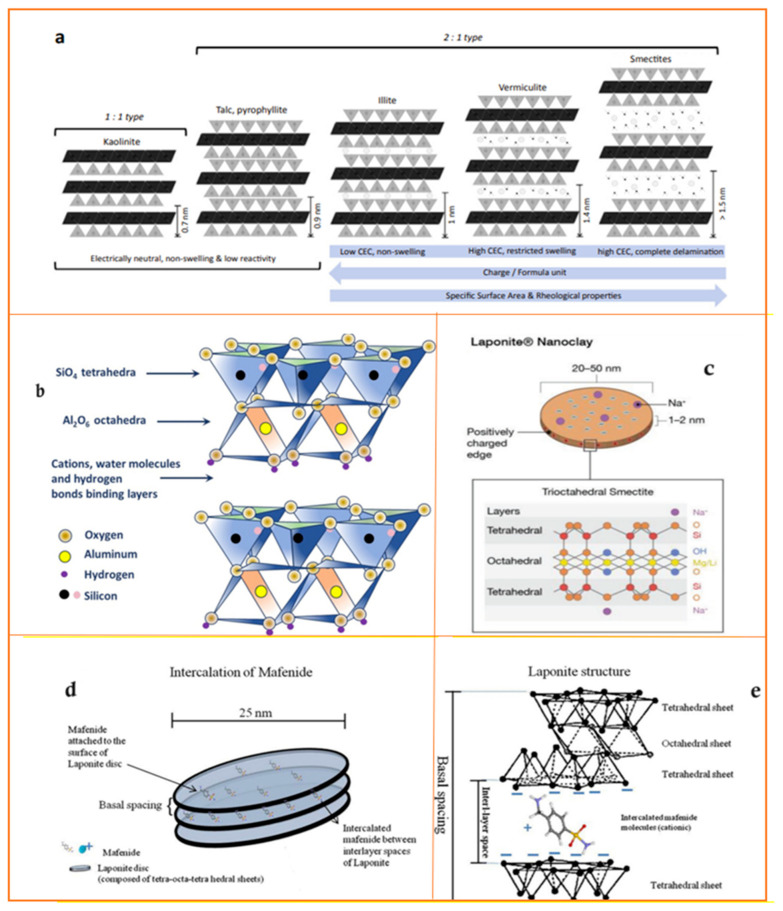
(**a**) The reactivity of clays primarily depends on their swelling capacity. Kaolinite (belonging to the 1:1 clay family) and talc and pyrophyllite (members of the 2:1 clay family) have no structural charges, making them non-swelling with low adsorption capacity. Vermiculite and illite, despite having a high layer charge that limits their swelling and gelling tendencies, possess relatively high surface area and cation exchange capacity. Smectites, with their relatively low layer charge, can completely dissociate in water, leading to unique rheological/gel-forming properties and surface reactivity [40]. (**b**) Structure of kaolinite [37]. (**c**) Illustration of the structure and composition of Laponite RD nanoclay [36]. (**d**) Schematic view of nanosized LAPONITE disks and inter-layer space between these disks; (**e**) the chemical structure of LAPONITE disks and intercalation of cationic ions and drugs (e.g., mafenide) between the inter-layer space [37].

Different types of nanoclays, including kaolinite, talc, pyrophyllite, illite, vermiculite, smectites, halloysite nanotubes, and sepiolite, each have unique structural and compositional characteristics, making them suitable for various biomedical applications, especially in TE for bone regeneration (Table 1 and Figure 3) [36,37,38,39,40].

Nanoclays significantly aid bone regeneration through mechanical reinforcement, bioactivity enhancement, and the controlled delivery of drugs and growth factors. By improving the mechanical properties of hydrogels and scaffolds, nanoclays make these materials more suitable for supporting bone tissue. The release of ions such as magnesium, sodium, lithium, and silicon from nanoclays promotes osteogenic differentiation and bone tissue formation. Additionally, nanoclays can sequester and release therapeutic molecules, ensuring controlled and sustained delivery essential for effective bone healing [36,37,38,39,40,41,42].

Zhang YD et al. (2024) underscore the potential of nanoclays in bone regeneration through in vivo studies in animal models. [43]. Laponite RD bioceramic constructs enhance bone formation without significant toxicity or inflammation in animal models [36]. Research shows that nanoclay-reinforced hydrogels, like those with Laponite RD, can promote new bone formation even without growth factors due to their superior mechanical and bioactive properties [36,37,38,39,40,41,42,43,44]. Hybrid scaffolds combining polymers and nanoclays, such as poly(glycerol sebacate)/Laponite RD, poly(ɛ-caprolactone)/Laponite RD, and poly(glycerol sebacate)/organo-montmorillonite (Cloisite 30B) [6], also demonstrate substantial improvements in bone regeneration by supporting osteogenic differentiation and new bone tissue formation [36,37,38,39,40,41,42,43,44,45,46].

**Table 1:** The structural and chemical characteristics of various nanoclays utilized in BTE.

Structure of Clay Particles	Nanoclay	Chemical Formula/Nanoclays/ Materials Involved	Layers	Species	Animal Model	Finding Indicated	CEC ^1^ (meq/100 g)	Particle Size (nm)	Reference
	MMT ^2^	Na_m_(Al_2_.-mMg_m_)Si_4_O_10_(OH)_2_·nH_2_O	2:1	Smectites			~80–150	~80–300 diameter ~1 thickness	[37,47,48]
Layered		MeGC-MMT hydrogel			Nude mice (8–12 week)	Satisfactory results only by applying the material itself			[36,49]
		PDLA - 2.5% MMT (*w*/*w* rhBMP-2)			Female mice Balb/C	Inconclusive results: 1.Comparable to control 2.Higher bone formation with rhBMP-2			[36]
Layered	Kaolinite	[Si_2_Al_2_O_5_(OH)_4_·nH_2_O (n = 0 or 4)	1:1				2	~50 to 600 &internal diameters ~2 to 20	[37,50]
Layered	Halloysite	Al_2_Si_2_O_5_(OH)_4_·nH_2_O	1:1	Serpentine Kaolinite			~10	Nanotube diameter of ~50, lumen of ~15, length of ~1 mm	[37,40]
		HNT/Ge/MA hydrogel			Sprague–Dawley rats	One regeneration improved with the presence of HNTs			[36]
Layered	Bentonite	(Na,Ca)_0.33_(Al,Mg)_2_(Si_4_O_10_)(OH)_2_·nH_2_O	2:1	Smectites			~70–110	~100–500 diameter ~1 thickness	[40,47,48]
Layered	Laponite R	Nah(Mg_3-h_Li_h_) Si_4_O_10_(OH)_2_.nH_2_O	2:1	Smectites			~4–40	~25–30 diameter ~1 thickness	[37,40]
		Laponite RD bio ceramic			Female rats and mature male pigs	1. No obvious toxicity 2. A totally healed bone lesion			[36]
		1.Laponite RD functionalized TBG 2.Laponite RD + alginate gels			Nude mice	1. Osteoconduction due to BMP-2, not clay-related 2. Gels able to localize BMP-2 to boost bone formation			[36,51]
		PEG4K-Laponite RD scaffolds (w/wo ROB)			12 week-old male Sprague–Dawley rats	1. Scaffolds + ROB stimulate new bone formation 2. Better osteogenic properties for scaffolds + ROB			[36]
		Hyaluronic-bisphosphonate hydrogel/Laponite RD/BMP-2			MF-1 wild type mice	HABP + Lap + BMP-2 scaffolds presented synergistic effects, resulting in major bone induction in contrast to all controls			[36,52]
		Gelatin-derived graphene/Laponite RD (GL-powder) BMP-9			Athymic nude mice	GL-powder able to enhance BMP9-induced ectopic bone formation from MSCs in comparison with BMP-9 alone			[36,53]
		3D-Scaffold: poly(glycerol sebacate) (PGS)/Laponite RD			Mice	1. From day 3 inflammatory infiltration at interfaces and at day 6 within scaffold 2. After day 21 degradation without inflammation			[36,54]
Layered	Illite	(K,H)Al_2_(Si,Al)_4_O_10_(OH)_2_·XH_2_O	2:1	Illite			15	0.075 μm, 0.3 μm, 1.2 μm (trimodal distribution)	[37,50,55]
Layered	Rectorite	(Na,Ca)Al4(Si,Al)_8_O_20_(OH)_4_·2H_2_O	1:1	Rectorite			~20–50	~200–300 diameter ~1–2 thickness	[37,56]
Fibrous	Palygorskite	(Mg,Al)_2_Si_4_O_10_(OH)·4(H_2_O)		Attapulgite			~4–40	25~30 diameter ~1 thickness	[37,40]

^1^ Cation exchange capacit. 2 Montmorillonite.

Future research should optimize nanoclay formulations and dosages, explore interactions with different polymers, and conduct comprehensive in vivo studies to realize their full clinical potential. Developing multifunctional scaffolds incorporating nanoclays and other nanomaterials could further advance TE’s capabilities in treating bone disorders.

#### 2.1.5. Zirconia

Zirconia, or ZrO_2_, recognized for its chemical stability, biocompatibility, and mechanical strength, attracts interest in regenerative medicine, especially in BTE [57]. It supports osteoblast proliferation and differentiation without adverse reactions and is considered osteo-inductive [58]. Kundu et al. created an organic-inorganic hybrid composite enhanced with ZrO_2_ NPs, achieving an ideal scaffold for BTE with comparable mechanical strengths and porosities to human cancellous bone and exhibiting antibacterial properties [59].

Research by Jayasuriya et al. assessed chitosan composite scaffolds hybridized with nano-HA, nano-zirconia (nZrO; ZrO_2_), and nano-calcium zirconate (nCZ; CaZrO_3_), finding that the addition of bio-ceramic powders improved mechanical strength, cell proliferation, and cell spreading, indicating a bright future for Zr-based bio-ceramic composites in bone TE [60].

Additionally, ZrO_2_ ceramics are employed in dental, orthopedic, and bone repair applications, with nanostructured ZrO_2_ and films/coatings demonstrating the ability to induce bone growth and exhibiting antibacterial properties without toxicity or immunogenicity concerns [61,62]. Li et al. (2016) reported on the successful surface modification of CFR-PEEK with bioactive ZrO_2_, enhancing cellular adhesion, proliferation, and osteogenic differentiation, suggesting its use in orthopedic implants [63].

Studies underscore Zr and ZrO_2_’s potential in enhancing the physical, chemical, and biological properties of scaffolds for BTE, offering innovative solutions for improved bone regeneration and implant integration [59,60,61].

#### 2.1.6. Bioactive Glass (BG) NPs

Bioactive nanoglass, especially those composed of a blend of HA and BG NPs, is emerging as a promising material for bone regeneration in TE [64]. Created using advanced sol–gel techniques, they form bio-nanocomposites that combine the bioactive properties of the NPs with the structural benefits of a porous matrix, such as alginate [65].

Highly effective due to their nanometric scale, these nanoglass composites enhance surface area and reactivity, enabling faster bone-like apatite formation when immersed in simulated body fluid (SBF). This is essential for creating a material that can integrate effectively with natural bone, supporting the growth and attachment of bone cells [66]. The bioactive glass component is noted for its osteoprotective capability, forming a biologically active silica-rich layer that facilitates bone tissue formation and helps the material bond to living tissues upon implantation [67,68].

Furthermore, integrating these NPs into an alginate matrix allows for the production of scaffolds that are not only biocompatible but also possess the necessary mechanical strength to maintain structural integrity until the new tissue can fully form and replace the scaffold material. Designed with an interconnected porous structure, these scaffolds are ideal for cell infiltration and vascularization, critical for effective TE [65].

Overall, nanoglass based on HA and bioactive glass represents a significant advancement in materials for bone regeneration, offering a combination of rapid bioactivity, excellent mechanical properties, and superior biocompatibility, which are key for successful bone repair and regeneration in clinical applications [69].

### 2.2. Metal NPs

Metal-based NPs, including those made from gold, silver, iron, aluminum, nickel, copper, zirconium, and magnetic materials, play a crucial role in advancing BTE [70]. Silver and copper NPs exhibit strong antibacterial properties, preventing infections at bone injury or surgical sites and creating a safe environment for bone healing [71]. Gold and copper NPs promote the osteogenic differentiation of stem cells into osteoblasts, essential for new bone tissue formation [72,73]. Additionally, these NPs enhance cell proliferation, with gold and copper accelerating bone tissue formation and repair [71,72,73]. Copper NPs also promote angiogenesis, the formation of new blood vessels, ensuring that regenerating tissues receive the necessary nutrients and oxygen [74]. Zirconium and aluminum NPs improve the mechanical properties of scaffolds, providing the strength and durability needed to support bone regeneration and withstand physiological loads [75]. Furthermore, some metal NPs facilitate controlled drug delivery, allowing precise administration of therapeutic agents like growth factors or antibiotics directly to the bone regeneration site, significantly improving treatment efficacy. Together, these diverse actions of metal NPs significantly contribute to the progress in BTE [76]. Applications of metal nanomaterials for BTE continue to expand, demonstrating their critical role in enhancing the effectiveness of treatments and advancing the field.

#### 2.2.1. Gold NPs

The utilization of gold nanoparticles (AuNPs) has garnered significant interest for various biomedical applications, including drug delivery, cell targeting, biosensing, TE, and notably, BTE [77,78]. Key advantages of AuNPs in this field are highlighted by their low toxicity, antibacterial properties, and high biocompatibility, with their inert gold core being a central feature despite some uncertainties regarding their cellular effects [79,80]. Studies have indicated that AuNPs’ cytotoxicity may vary based on factors like size, concentration, surface chemistry, and cell type, often being dose-dependent and associated with cellular membrane damage, content leakage, and reactive oxygen species generation, which can disrupt the balance between pro-oxidant and antioxidant reactions within cells [81].

Further exploration into AuNPs’ role in BTE reveals their potential in enhancing osteogenic differentiation of stem cells through various pathways, such as Wnt/β-catenin, ERK/MAPK, and p38/MAPK pathways [82,83]. Innovative approaches, like the development of hybrid hydrogels incorporating AuNPs, have shown promising results in promoting bone regeneration in vivo. Conversely, the use of gold nanorods (AuNRs) in this context is less explored due to challenges like toxicity from surfactants used in their synthesis and issues with aggregation. Nevertheless, surface modification strategies, such as using natural polymers for stabilization, have demonstrated effectiveness in mitigating these issues and enhancing biocompatibility for drug delivery purposes [84].

Additionally, the potential of bone mesenchymal stromal cells (BMSCs) in tissue regeneration is highlighted, with microRNAs serving as a novel epigenetic regulation mechanism that can modulate gene expression post-transcriptionally [83]. The use of ultra-small AuNPs as vehicles for miRNA delivery suggests their efficacy in enhancing osteogenic differentiation by protecting and delivering miRNAs to target sites, thus promoting bone tissue regeneration [83].

In Table 2, different shapes available for AuNPs are categorized, including nanoclusters, nanoshells, nanobranches, nanotriangles, nanocubes, nanohexagons, nanopentagons, nanorods, nanospheres, nanocages, and nanostars.

#### 2.2.2. Silver (Ag) NPs

There are complexities and advancements in utilizing silver and silver NPs within orthopedic and TE applications, emphasizing the critical balance between antimicrobial efficacy and cytotoxicity to achieve optimal outcomes in bone defect healing and implant success [60].

The role of Ag, both in metallic form and as silver salts, in enhancing the antibacterial properties of implants. Silver’s mechanism of action involves penetrating bacterial cells, interfering with DNA processes, and ultimately leading to bacterial death. Advantages of Ag-based scaffolds include their potent antimicrobial activity, good cytocompatibility, and supportive role in osteogenesis and cell proliferation, though the therapeutic window is narrow due to the cytotoxicity at high concentrations [60].

Xu et al. (2016) demonstrate the successful incorporation of silver and strontium into HA/chitosan scaffolds, significantly reducing bacterial presence and suggesting a dual function of osteoinductivity and antibacterial capability. The broader application of AgNPs across various biomedical fields is also detailed, including their role in wound dressings, drug delivery, and as coatings for medical instruments, largely owing to their antimicrobial properties [85].

The impact of AgNPs on cellular processes, particularly in the context of wound healing and bone regeneration, is examined through their interactions with human mesenchymal stem cells (HMSCs) and their role in promoting osteoblast differentiation. The document underscores the necessity of balancing the benefits of AgNPs against their potential cytotoxic effects, which are concentration and exposure duration-dependent [86]. Emerging research, including that by Akturk et al. (2020) and Kumar Saini et al. (2019), explores the development of nanocomposite materials incorporating AgNPs for scaffold applications, aiming at enhancing antibacterial properties without compromising cell viability and support for BTE [87,88]. Hasan et al. (2018) and Wang et al. (2015) further validate the efficacy of these silver-doped materials in achieving significant antimicrobial activity, thereby contributing to the broader effort to mitigate the risks of implant-related infections and enhance bone regeneration outcomes [89,90].

#### 2.2.3. Iron NPs

In BTE, iron-based NPs are promising for scaffold construction due to their ability to replicate bone constituents, providing mechanical strength and a porous structure for tissue integration [60]. De Santis et al. (2015) introduced a 3D scaffold with a PCL matrix reinforced with iron-doped HA(FeHAp) NPs, enhancing bone marrow stem cell (BMSC) loading and proliferation under a static magnetic field [91]. However, metallic scaffolds, including iron, raise concerns about tissue contamination from metal ion release, potentially necessitating implant removal. Despite not being naturally present in bone, iron is crucial for bone formation, suggesting its suitability for scaffolds [92]. The shape of iron NPs affects cell toxicity, with surface modifications proposed to enhance biocompatibility. To address rapid scaffold degradation and cytotoxicity, iron–tungsten (FeW) alloys have been developed to slow degradation rates while maintaining biodegradability and non-toxicity [93]. He et al. (2016) created porous Fe/FeW double-layered scaffolds, finding that slower-corroding scaffolds have less impact on cell viability, indicating their potential for BTE applications due to optimized degradation rates and biocompatibility [86].

#### 2.2.4. Copper NPs

Copper (Cu) is recognized as a critical trace element for the human body, essential for several biochemical and physiological functions, including the generation of Cu-proteins with enzymatic activities, bone metabolism regulation, and influencing the nervous system’s balance [94,95]. Its unique properties, such as catalytic abilities, antibacterial, and antifungal activities, along with its role in stimulating collagen fiber deposition and angiogenesis, have made it a focal point of interest in the field of BTE. Cu’s capability to induce mesenchymal cell differentiation into the osteogenic lineage highlights its importance in bone health, requiring the catalytic activity of lysyl oxidase, a Cu-dependent enzyme. This enzyme mediates the biosynthesis of collagen, elastin, and keratin and regulates the deposition of calcium and phosphorus in bones [96,97]. The deficiency of Cu is linked to reduced bone mass, diminished mechanical strength, and increased risk of fractures, attributed to the compromised function of osteoblast cells, emphasizing the necessity of adequate Cu supplementation for bone formation [97].

The burgeoning interest in CuNPs stems from their enhanced physical activities, allowing for reduced dosage, and their recognized potential as antimicrobial agents and osteoporosis treatments [97,98]. CuNPs have shown efficacy in bone mineralization, enhancing the adhesion and proliferation of osteoblast cells, and the synthesis of bone connective tissue [98,99]. Despite their benefits, CuNPs have been reported to induce intense inflammatory responses and cellular toxicity, influenced by factors like size, concentration, solubility, and biodistribution [100]. Mitigation strategies involve coating CuNPs with biocompatible materials to enhance stability and biocompatibility and prevent surface oxidation [101].

Innovative applications, such as the PCL/RGO-Cu nanocomposite, have shown sustained copper ion release, enhancing angiogenic activity, and promoting significant osteogenic activity in pre-osteoblast cells, along with improved antibacterial effectiveness, positioning it as a suitable candidate for BTE [102]. Similarly, the CS/nHAp/nCu–Zn scaffold, by incorporating Cu–Zn alloy NPs, has demonstrated increased swelling, reduced degradation, and enhanced antibacterial and osteoproliferative properties, suggesting its utility in bone formation without toxicity towards osteoprogenitor cells [103].

#### 2.2.5. Zirconium NPs

Zirconium (Zr), a natural element found in trace levels in bones, is acknowledged for its osteoinductive and biocompatible properties. It enhances osteoblast proliferation and differentiation through the BMP/SMAD signaling pathway [60]. Due to its high strength, corrosion resistance, magnetic sensitivity, and low cytotoxicity, Zr is a promising candidate for biomedical applications [57,104]. Maghsoudlou et al. (2020) developed a biodegradable nanocomposite incorporating Zr NPs with chitosan, HA, and wollastonite (WS) through a freeze-drying method, showing enhanced mechanical properties and biocompatibility for BTE [105].

In medical and dental implants, Zr’s efficiency is well-established, especially noted for its osseointegration capabilities and chemical stability, making Zr-containing materials and coatings suitable for bone implants due to their ability to form apatite structures [106].

#### 2.2.6. Aluminum NPs

Aluminum and its derivatives, especially in oxidized forms like Al_2_O_3_, offer significant benefits in BTE. These include mechanical strength, improved cell adhesion and proliferation, and enhanced scaffold stability, making aluminum a valuable material for bone regeneration scaffolds [107]. Additionally, aluminum and its oxides contribute to the mechanical strength and fracture resistance of scaffold materials. Porous ceramic coatings on these forms enhance osteointegration, providing resistance to long-term implantation failure [86,108]. Aluminum oxide and its ionic forms have been utilized to support cellular proliferation and the activity of multinucleated osteoclastic cells, indicating low cytotoxicity and biodegradability in materials like Ca_2_Al_2_SiO_7_ [86,108].

Chen et al. (2017) investigated the osteogenic effects of macrophage cell cultures on anodized aluminum with a honeycomb morphology. They found that cell spreading, autophagy gene expression, and osteogenic factor release were influenced by nanopore size. Structures with 100–200 nm nanopores showed reduced pro-inflammatory cytokine gene expression and ROS levels. Meanwhile, 50 nm pores up-regulated mineralization, osteopontin, and collagen 1 expressions, indicating that nano-porous structures can enhance cell proliferation but may pose viability risks when pores are reduced to the nanoscale [109].

Karunakaran et al. (2014) highlighted the comparative cytotoxicity of micro- and nano-SiO_2_ and Al_2_O_3_ on NIH 3T3 cells, noting that antioxidant activity was more pronounced at the nanoscale [110]. Park et al. (2008) demonstrated that biomimetic coating with calcium phosphate on alumina-based ceramics altered porosity depending on the deposition amount, emphasizing the importance of biomimetic materials in scaffold design over mere pore size [111].

Restrepo et al. (2015) investigated the viability of Vero cells in contact with hybrid carbon nanotube (CNT)/aluminum NP composites, finding an increase in cell viability as the proportion of CNTs decreased [112]. Yu et al. (2020) showed that Al_2_O_3_ in the form of Ca_2_Al_2_SiO_7_ scaffolds contributed to the formation of interconnected macropores essential for cell adhesion, nutrient flow, and proliferation. Increased proportions of Al_2_O_3_ in these scaffolds reduced degradation, offering a more stable environment for cell differentiation and bone regeneration [113].

#### 2.2.7. Nickel NPs

Nickel (Ni), a naturally occurring block “d” metal in human blood, has been recognized for its low toxicity at normal serum levels and its potential in BTE due to its dose-dependent toxicity [112]. Ni alloys are valued for their multifunctionality, high resilience, strength, low Young’s modulus, and shape memory effects, making them ideal for scaffolds with controlled porosity, size, shape, and surface chemistry to enhance tissue regeneration [60].

Kokorev et al. (2016) developed a porous TiNi scaffold using a self-propagating high-temperature method, demonstrating its ability to support mesenchymal stem cell (MSC) differentiation into bone tissue over 28 days. This scaffold also showed potential for vascularization and nutrient delivery, crucial for successful bone regeneration, emphasizing the role of angiogenic growth factors like VEGF and PDGF in promoting endothelial cell proliferation and migration [114].

Anu Priya et al. (2015) found that Ni^2+^-doped nano-hydroxyapatite (nHAp) enhances MG-63 cell viability without significant toxicity. RT-PCR analysis revealed that Ni^2+^-doped nHAp up-regulated Runx2 expression, critical for osteoblastic differentiation, via the Ca^2+^-dependent Wnt5 signaling pathway, indicating Ni^2+^’s role in bone formation processes [115].

Song et al. (2016) explored a composite structure using nickel foam, graphene oxide, and polypyrrole with HAp through a layer-by-layer assembly strategy. These scaffolds enhanced MC3T3-E1 cell proliferation and adhesion, suggesting the composite’s effectiveness in supporting bone cell growth and adhesion. This research indicates that while Ni might not be directly incorporated into scaffold structures, it can act as a topologic director for layer adaptation, improving shape, structural morphology, and surface hydrophobicity for better cellular interactions [116].

Nickel and its compounds enhance the design and functionality of scaffolds for BTE, leveraging their unique physical properties and biological interactions to support bone regeneration and TE applications [117].

#### 2.2.8. Magnetic NPs

Magnetic NPs offer a multifaceted approach to BTE, from enhancing scaffold properties and stem cell therapies to providing innovative treatments for bone tumors [118].

Iron and its derivatives, including iron oxide NPs, exhibit unique magnetic properties, particularly notable in magnetite (Fe_3_O_4_) and maghemite (γ-Fe_2_O_3_) forms, due to their superparamagnetic behavior. These NPs have catalytic capabilities and have been developed into various composites for biomedical applications, including drug delivery and cancer therapy, due to their ability to respond to external magnetic fields [60,119].

Significant in the advancement of BTE, these NPs have been incorporated into scaffolds to enhance osteogenic differentiation and cellular responses, crucial for bone formation and the treatment of bone-related diseases. Kim et al. (2014) demonstrated the benefits of incorporating magnetic NPs into PCL scaffolds, showing improved hydrophilicity, mechanical stiffness, and mineral induction, which are advantageous for bone regeneration [120].

Furthermore, magnetic NPs have been utilized for in vivo labeling and tracking of mesenchymal stem cells (MSCs), offering a new avenue for regenerative medicine, particularly in stem cell therapy [120,121]. The development of magnetic scaffolds, through the functionalization of nanoparticle surfaces, aims to prevent aggregation and enhance biocompatibility, contributing to the dispersibility and effectiveness of these scaffolds in BTE [119].

The application of magnetic NPs also extends to the treatment of bone tumors through hyperthermia therapy, leveraging magneto-thermal activation to eradicate tumor cells while sparing normal tissues [122]. Innovative strategies incorporate these NPs into bioactive glasses or polymer scaffolds, although challenges remain in optimizing the magnetism and magneto-thermal effects within biomaterial matrices [123,124].

Additionally, the integration of magnetic NPs with HAp and chitosan matrices has been shown to enhance bioactivity and osteoblast proliferation, further underscoring the potential of these NPs in bone regeneration [125].

### 2.3. Carbon-Based NPs

**Figure 4:**
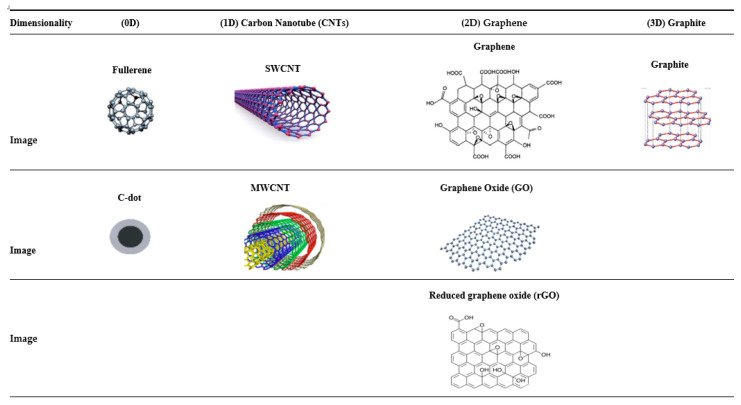
The various dimensionalities of carbon-based NPs and their structures are illustrated, including 0D fullerenes, 1D single-walled and multi-walled carbon nanotubes (SWCNT, MWCNT), 2D graphene oxide and reduced graphene oxide, and 3D graphite and diamond.

Carbon-based NPs have emerged as promising agents in the field of bone regeneration due to their unique physicochemical properties. Their high surface area, biocompatibility, and ability to facilitate cellular interactions make them ideal for enhancing osteogenesis [126]. These NPs, including graphene, carbon nanotubes, and fullerenes (Figure 4), have shown potential for promoting stem cell differentiation and bone tissue formation [127]. Recent studies highlight their effectiveness in delivering osteoinductive agents and providing scaffolding for bone growth, positioning carbon-based NPs as a pivotal component in the future of TE for bone regeneration.

#### 2.3.1. Zero-Dimensional Carbon-Based Nanomaterials for BTE

Zero-dimensional (0D) carbon-based nanomaterials primarily consist of carbon dots (C-dots), fullerene, and nanodiamonds (NDs) [126].

##### Carbon Dots (C-Dots)

C-dots are emerging as promising agents for bone regeneration [127]. These NPs are less than 10 nm in size, highly stable, and exhibit bright fluorescence, making them suitable for both therapeutic and diagnostic applications [127,128]. Studies have shown that C-dots specifically bind to developing and regenerating bones [129]. In vivo experiments with zebrafish revealed that C-dots were deposited rapidly and selectively in bones undergoing growth and repair. This specificity is crucial for their potential use in targeted drug delivery systems for bone diseases [130,131].

C-dots’ natural fluorescence and strong bone-binding properties position them as excellent candidates for theragnostic applications [132]. They can serve as both imaging agents for early detection of bone diseases and as drug carriers to deliver therapeutic agents directly to bone tissues, minimizing systemic side effects [133,134].

##### Fullerene (C60) for BTE

Fullerene, known for its unique cage-like structure composed of 60 carbon atoms, holds significant promise in the field of BTE due to its exceptional physicochemical properties. Fullerenes exhibit a high degree of symmetry and stability, which contributes to their ability to interact with biological systems effectively. Recent research has highlighted several potential applications of fullerenes in bone regeneration and repair [135].

One of the primary advantages of using fullerenes in BTE is their ability to reduce the formation of reactive oxygen species (ROS) [136]. Excessive ROS can lead to oxidative stress, which negatively impacts bone health by promoting osteoclast differentiation and bone resorption. Studies have shown that fullerene derivatives, such as C_60_(OH)_30_ and C_60_(OH)_16_AMBP, exhibit strong affinity for HA, the mineral component of bone. These derivatives can significantly reduce HA mineralization, thereby decreasing the crystal growth rate and potentially limiting bone degradation [137,138].

Additionally, fullerenes’ antioxidant properties play a crucial role in mitigating oxidative stress-related damage in bone tissues. By scavenging free radicals, fullerenes can protect bone cells from oxidative damage, promoting healthier bone regeneration. This characteristic is particularly beneficial in conditions like osteoporosis, where bone density and strength are compromised due to increased oxidative stress [135].

Innovative research has demonstrated the potential of fullerenes to support osteoblast adhesion and growth. For example, aligned fullerene C60 nanowhiskers have been used as scaffolds for osteoblast cultures, showing promising results in directing cell growth and promoting bone tissue formation. These nanostructures provide a favorable environment for bone cells, enhancing their proliferation and differentiation, which are critical for effective bone regeneration [139].

Despite these promising findings, the application of fullerenes in BTE is still in its early stages. Further research is needed to optimize the functionalization of fullerenes to enhance their biocompatibility and effectiveness in clinical settings. Future studies should focus on understanding the long-term effects of fullerene-based materials on bone health and their potential integration into existing bone regeneration therapies [135].

##### Nanodiamonds (NDs) for BTE

NDs have garnered significant attention in BTE due to their unique properties, including biocompatibility, high surface area, chemical stability, and excellent mechanical properties. These characteristics make NDs highly suitable for developing advanced scaffolds that support bone regeneration and repair [140].

One of the primary advantages of nanodiamonds is their ability to provide a three-dimensional (3D), porous structure that closely mimics the native bone environment. This structure is crucial for facilitating cell proliferation and differentiation, which are essential processes for effective bone healing [140].

NDs possess highly functional surfaces that can be modified to enhance their interaction with biological systems [141]. This functionalization allows NDs to deliver therapeutic agents directly to bone tissues, thereby reducing systemic side effects and improving the efficiency of bone regeneration treatments [142].

Recent studies have highlighted the potential of NDs to reduce oxidative stress, which is a significant factor in bone degradation [143]. By scavenging free radicals, NDs can protect bone cells from oxidative damage, thereby promoting healthier bone tissue formation. This antioxidative property is particularly beneficial in treating conditions such as osteoporosis, where bone density and strength are compromised [144].

Innovative research has demonstrated the ability of nanodiamond-based scaffolds to support osteoblast adhesion and growth [145]. For example, NDs have been used to create scaffolds that provide a favorable environment for bone cells, enhancing their proliferation and differentiation. These properties are critical for developing effective bone regeneration therapies [146].

Future studies should focus on understanding the long-term effects of ND-based materials on bone health and exploring their integration into existing bone regeneration therapies.

#### 2.3.2. One-Dimensional Carbon-Based Nanomaterials for BTE

##### Carbon Nanotubes (CNTs)

Recent advancements in nanotechnology have highlighted CNTs as an advantageous material for enhancing bone regeneration [147]. CNTs are divided into two types based on their cylindrical layer count: single-walled CNTs (SWCNTs) and multi-walled CNTs (MWCNTs) [126]. With their large surface area and exceptional mechanical properties, CNTs play a crucial role in promoting osteoconduction within composites [3]. Research has demonstrated that scaffolds containing CNTs improve the adhesion, proliferation, and differentiation of osteoblasts—vital processes for bone tissue development [148]. The interaction between CNTs and cells is primarily attributed to their surface features, which can be customized to enhance interactions with bone cells, creating an environment favorable for bone healing [149].

The mechanical characteristics of CNTs closely resemble those of natural bone, providing the necessary support and stability for bone tissue formation [150]. Additionally, their chemical composition allows for the effective adsorption and retention of growth factors, increasing the osteogenic potential of scaffolds. These qualities collectively contribute to the development of scaffolds that significantly aid in bone regeneration [148,151].

**Figure 5:**
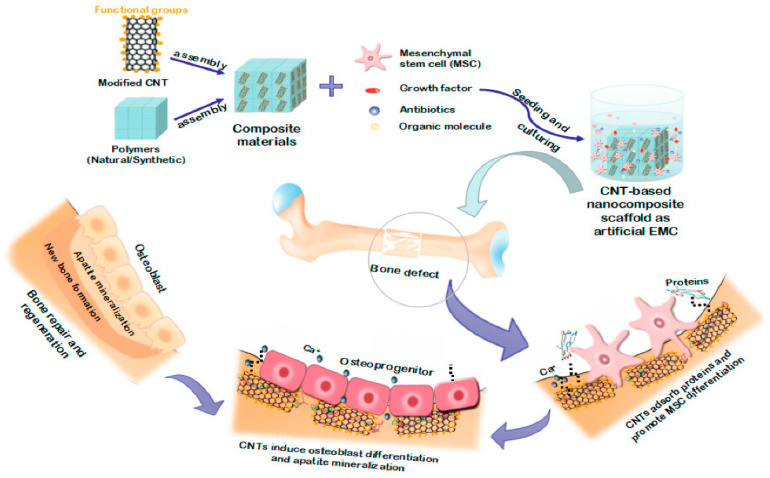
Diagram showing the role of CNTs as scaffold composites in BTE and regeneration, reprinted from Ref. [147].

Studies on the biosafety of CNTs have shown minimal degradation and adverse reactions in biological systems, indicating that properly processed and applied CNTs do not cause long-term inflammation or carcinogenic effects, thus confirming their suitability for bone regenerative medicine (Figure 5) [147]. The combination of CNTs with HA, a key bone mineral, has led to the development of novel composite materials for bone implants [147,152,153]. These composites, incorporating chitosan or its amphiphilic derivative with CNTs through a wet precipitation method, not only mimic the ECM but also act as a scaffold for controlled drug release [154].

#### 2.3.3. Two-Dimensional Carbon-Based Nanomaterials for BTE

##### Graphene and Graphene Oxide (GO)

Graphene-based NPs, particularly GO, have garnered significant attention in BTE due to their unique properties that facilitate bone regeneration processes, offering substantial advancements in the field.

Graphene is a two-dimensional carbon nanomaterial known for its exceptional mechanical properties, including high tensile strength and flexibility. GO, a derivative of graphene, introduces various oxygen-containing functional groups such as hydroxyl, carboxyl, and epoxy. These functional groups enhance GO’s dispersibility in aqueous solutions and improve its interaction with biological molecules, making it highly suitable for biomedical applications [154,155].

Studies have demonstrated that GO and its composites exhibit excellent biocompatibility and can significantly support the proliferation and differentiation of osteogenic cells. For instance, composites made of chitosan and GO with a higher GO content (2.5–6 wt%) have shown increased cell proliferation and lower cytotoxicity, providing a conducive environment for osteoblast growth and differentiation. This is crucial for effective bone regeneration as it supports the attachment, proliferation, and differentiation of MSCs [156,157].

In vitro experiments indicated that MSCs retained their viability and proliferative capabilities when cultured on GO-coated surfaces. For example, goat adipose-derived mesenchymal stem cells (AdMSCs) adhered well to GO NPs, proliferated, and maintained their stem cell properties. These cells also underwent osteogenic differentiation without the need for additional chemical inducers, suggesting that GO itself can act as an osteoinductive material [158].

The incorporation of GO into scaffold materials significantly enhances their mechanical properties. Scaffolds made from materials like PCL reinforced with GO have shown increased tensile strength, elongation, and Young’s modulus compared to pure PCL scaffolds. This mechanical enhancement is essential for supporting the structural integrity of scaffolds in vivo, ensuring they can withstand physiological loads and stresses [159,160].

GO’s ability to promote osteoconductivity and osteoinductivity has been well documented. Graphene-based scaffolds have been shown to support the osteogenic differentiation of hMSCs. In one study, 3D graphene foams used as culture substrates for hMSCs maintained stem cell viability and promoted osteogenic differentiation due to the large surface area and conducive microenvironment provided by the graphene scaffold [161].

Moreover, combining GO with HA enhances the scaffold’s properties further. GO-reinforced HA composites not only improve mechanical strength but also enhance biological performance, such as cell viability and proliferation. This combination offers both structural support and biological cues essential for effective bone regeneration [162,163]. Elkhenany et al. (2015) demonstrated in a study that the implantation of low oxygen content graphene (LOG) NPs mixed with MSCs into rat tibial bone defects resulted in improved bone formation and increased mineralization. The combination of LOG NPs and MSCs led to significant healing of bone defects, highlighting the potential of GO in practical bone regeneration applications [158].

##### Reduced Graphene Oxide (rGO)

rGO has emerged as a significant material in the field of bone regeneration due to its remarkable properties and potential to enhance the efficacy of osteoconductive scaffolds. The integration of rGO in BTE scaffolds has been shown to improve mechanical properties, promote osteoblast adhesion, and enhance osteogenic differentiation.

rGO-enhanced scaffolds exhibit improved mechanical properties, such as increased elastic modulus and fracture toughness. For instance, HAp and β-tricalcium phosphate (β-TCP) composites with rGO have demonstrated enhanced mechanical strength, addressing the typical limitations of these materials, which include inadequate mechanical properties and insufficient osteoconductivity. The presence of rGO improves the mechanical resilience of these composites, making them more suitable for load-bearing applications in BTE [164,165,166].

Studies have shown that rGO can significantly promote the adhesion and proliferation of osteoblasts. For example, rGO/HAp composites have been found to enhance the osteogenesis of preosteoblastic MC3T3-E1 cells, fostering new bone formation and promoting spontaneous osteodifferentiation [166]. The high surface area and unique surface properties of rGO facilitate better protein adsorption and stability, which are crucial for cell attachment and proliferation. These properties also contribute to the increased hydrophilicity of the scaffold surfaces, further enhancing cell-scaffold interactions [167].

The integration of rGO into scaffolds has been shown to support the differentiation of MSCs into osteogenic lineages [168]. rGO-modified scaffolds provide a conducive environment for stem cell differentiation by mimicking the natural ECM and delivering necessary biochemical cues. This is particularly evident in rGO-doped PLGA/HAp nanofiber scaffolds, which promote the proliferation and osteogenic differentiation of human MSCs, demonstrating significant potential for reconstructing large bone defects [169,170].

Despite the promising results, several challenges remain in the application of rGO in BTE. These include concerns related to cytotoxicity, biodistribution, and immune responses. However, modifications to the rGO surface, such as functionalization with biocompatible molecules, have been shown to mitigate these issues, enhancing its biocompatibility and reducing cytotoxic effects [170].

Future research should focus on long-term in vivo studies to evaluate the safety and efficacy of rGO-based scaffolds. Additionally, exploring the synergistic effects of combining rGO with other bioactive molecules or growth factors could further enhance the osteogenic potential of these scaffolds, paving the way for advanced bone regeneration therapies.

##### Three-Dimensional Carbon-Based Nanomaterials for BTE

Graphite and diamond, the most well-known 3D carbon-based materials, have been recognized since ancient times. These materials differ primarily in their crystal structures and properties. Despite their widespread applications, they were not directly studied as bone BTE scaffolds due to their lack of porosity. BTE scaffold materials need to have an adequate number of appropriate pores to support cell adhesion, proliferation, and osteogenic differentiation [171]. A technique was developed to fabricate porous 3D structures by carbonizing polyacrylonitrile. The resulting carbonized 3D structure (cPAN) featured pore diameters ranging from 75 to 100 μm and exhibited a graphitic structure akin to CNTs. In comparison to glass and CNTs used as BTE scaffolds, cPAN showed superior performance in terms of cell viability, proliferation, and osteoinduction. This was demonstrated through studies on cell morphology, alkaline phosphatase (ALP) activity, runt-related transcription factor 2 protein expression, and calcium content in MSCs after 17 days of culture. The exceptional performance of cPAN was attributed to its enhanced adsorption of ECM proteins, such as fibronectin, which potentially stimulate osteogenesis signaling pathways in cells. Additionally, the efficacy of 3D cPAN as a bone graft was confirmed by an in vivo study, where critical-sized calvarial defects in mice were fully repaired following the implantation of bone morphogenetic protein 2 (BMP-2)-loaded cPAN [171,172].

## 3. Organic NPs

Organic nanoparticles are highly valued in BTE for their biocompatibility, biodegradability, and customization for various biomedical uses. Organic nanoparticles in BTE are mainly categorized into synthetic and natural polymers, each with distinct advantages and challenges. This section focuses on the use of synthetic polymers [173].

### 3.1. Synthetic Polymers

Synthetic polymers provide numerous benefits, such as ample availability, straightforward fabrication and customization, high safety standards, and affordable costs. Their adjustable physicochemical and morphological features make them valuable for large-scale production and application [174,175]. The most frequently used synthetic polymers in biomedical applications include poly(α-hydroxyesters) such as poly(lactic acid) (PLA), poly(lactic-co-glycolic acid) (PLGA), polyethylene glycol (PEG), poly(propylene fumarate) (PPF), poly(ε-caprolactone) (PCL), and poly(glycolic acid) (PGA) [176]. However, these materials also have several limitations. Their hydrophobic nature impairs the loading of hydrophilic drugs or molecules and results in poor cell adhesion. They degrade through autocatalysis, leading to unpredictable degradation behavior. Additionally, their acidic degradation products can denature bioactive proteins and induce inflammatory tissue responses. Moreover, they possess a low capacity for loading therapeutic agents, restricting their diffusion within the polymer matrix [85,176,177,178]. Despite these challenges, various combinations of PLGA, PGS, PLLA, PEG, and PCL have demonstrated promising results in bone regeneration applications [178].

#### 3.1.1. Poly(Lactic Acid) PLA in Bone Regeneration

PLA is a synthetic, biodegradable polymer widely used in BTE due to its excellent biocompatibility, thermal stability, and favorable degradation profile. Approved by the US Food and Drug Administration (FDA) for various biomedical applications, PLA’s properties make it particularly suitable for creating scaffolds through fused deposition modeling (FDM) technology [179,180,181]. FDM is an additive manufacturing technique where PLA filaments are melted and extruded layer-by-layer to form three-dimensional structures. This method allows for precise control over scaffold porosity and pore size, which are crucial for supporting cell proliferation and differentiation, essential for bone regeneration [180,181].

In recent studies, PLA scaffolds with different pore sizes were fabricated using FDM to evaluate their physicochemical and biological properties. The scaffolds were designed with square pores of varying sizes and characterized using structural, chemical, mechanical, and biological assessments. The results demonstrated that, while the actual pore dimensions were slightly smaller than the predicted values, the process was reproducible with high precision. The FDM process was found to reduce the molecular weight and degradation temperatures of PLA, though it did not affect the polymer’s semi-crystalline structure [180].

Mechanical testing of the PLA scaffolds indicated that pore size did not significantly impact their mechanical properties, maintaining sufficient strength for handling during implantation. Sterilization by gamma irradiation ensured the scaffolds were non-cytotoxic, as confirmed by human bone marrow stromal cell (HBMSC) viability assays. The cells showed high viability and homogeneous distribution across the scaffolds, irrespective of pore size, after three and seven days of culture [180].

The application of PLA scaffolds in BTE is promising due to their biodegradability and ability to support cell attachment, proliferation, and differentiation [181]. The degradation products of PLA, primarily lactic acid, are metabolized by the body without causing toxicity, making PLA a safe material for clinical use [182]. Moreover, combining PLA with other materials, such as HA, provides a valuable tool in BTE by merging the biodegradability and favorable mechanical properties of PLA with the bioactivity of HAp. This combination creates an environment that closely mimics the natural bone structure and composition [183].

#### 3.1.2. Poly (Lactic-Co-Glycolic Acid) (PLGA)

PLGA has garnered significant attention in BTE due to its biocompatibility, adjustable degradation rates, and potential for modification. PLGA is synthesized from lactic acid and glycolic acid, with properties tailored by altering the ratio of these monomers. Generally, a higher lactic acid content results in slower degradation due to increased hydrophobicity [184,185].

These properties make PLGA an ideal carrier for bioactive molecules like growth factors, which are crucial for bone regeneration. Specifically, the article discusses the use of PLGA NPs for delivering BMP2, a key player in bone growth and repair [185].

The controlled delivery system of PLGA allows for a sustained release of BMP2, enhancing the osteoinductive properties necessary for bone regeneration. The encapsulation of BMP2 within PLGA NPs protects the protein from premature degradation and ensures its targeted delivery to the bone defect site. This targeted approach is critical as it maximizes the growth factor’s efficacy while minimizing potential side effects [185,186].

There are various strategies to optimize the encapsulation efficiency and release kinetics of BMP2 from PLGA NPs. These strategies ensure that the therapeutic potential of BMP2 is fully realized, promoting effective bone regeneration in clinical settings. Surface modifications ensure targeted delivery, while co-encapsulation with stabilizers protects BMP2 and stabilizes it within the NPs. Adjusting the polymer composition and particle architecture modulates PLGA’s degradation, enabling a sustained release of BMP2 for effective therapeutic outcomes [187].

Overall, PLGA NPs represent a promising technology for advancing BTE through enhanced delivery of osteogenic growth factors [184].

#### 3.1.3. Polyethylene Glycol (PEG)

PEGNPs, specifically enhanced with polyethyleneimine-PEG (PEI-PEG) coatings, are employed for effective delivery of BMP-2, a crucial factor in bone regeneration. The PEGylation of PEI considerably reduces the cytotoxicity typically associated with PEI, thereby enhancing the biocompatibility of the NPs. These NPs facilitate the sustained release and stabilization of BMP-2, maintaining its bioactivity, which is essential for promoting bone growth and repair. This targeted delivery system is particularly beneficial in BTE, as it ensures BMP-2 remains at the site of bone defects, providing a controlled release that is vital for effective bone regeneration. The integration of PEG into the nanoparticle design not only mitigates toxicity but also improves the delivery efficiency of BMP-2, making this approach promising for clinical applications in bone repair and regeneration [188].

#### 3.1.4. Poly(Propylene Fumarate) (PPF)

PPFNPs, particularly when combined with strontium-substituted hydroxyapatite (Sr-HA), have shown promising results in BTE. Jarrar et al. focused on PPF nanocomposite scaffolds incorporating Sr-HA to enhance their osteogenic potential. These scaffolds were designed to leverage the osteoinductive and osteoconductive properties of Sr-HA, which is known to promote bone formation and inhibit osteoclast activity, thereby enhancing bone regeneration [189].

The PPF/Sr-HA scaffolds were prepared with varying Sr concentrations in the Sr-HA particles, and their effects on bone cell activities were thoroughly investigated. These scaffolds supported better cell adhesion, proliferation, and osteogenic differentiation compared to scaffolds without Sr-HA, indicating the beneficial role of strontium in bone tissue scaffolds. The inclusion of Sr-HA not only improved the mechanical properties of PPF scaffolds but also enhanced their biological functionality, making them highly effective for applications in bone regeneration [189].

#### 3.1.5. Poly(ε-Caprolactone) (PCL) in Bone Regeneration

PCL is a highly versatile and biocompatible biodegradable polyester that has gained significant attention in the field of BTE [190]. PCL has strong mechanical properties that can handle stress after being implanted. The scaffold’s porosity is key for these properties, tissue penetration, and blood vessel growth. Larger pores reduce stiffness and improve cell and blood vessel infiltration [6].

Research shows that scaffolds with offset and gradient pore sizes are dense and porous, aiding cell infiltration in bone engineering. These gradients mimic natural bone, transitioning from dense cortical to porous cancellous bone, supporting cell movement, nutrient flow, and waste removal. Dense areas of the gradient scaffold offer strong support and maintain elasticity after stress. Studies found that larger pores in gradient scaffolds boost bone growth and mineralization by improving oxygen and nutrient supply [191,192,193,194].

Recent studies have focused on developing PCL scaffolds with gradient and offset pore architectures. These designs aim to mimic the hierarchical and heterogeneous structure of natural bone, which varies from the dense cortical bone to the porous cancellous bone [195,196]. The gradient pore size scaffolds, with gradually increasing pore sizes, provide a favorable environment for nutrient and oxygen diffusion, waste removal, and vascularization. This gradient structure supports better cell infiltration and differentiation, leading to more effective bone regeneration [193,194].

PCL scaffolds with various architectures have shown significant osteoconductive properties. Scaffolds with gradient pore sizes, which mimic natural bone architecture, exhibit superior bone regeneration compared to those with uniform pore sizes. In rodent models with critical-sized calvarial defects, these gradient scaffolds have resulted in substantial new bone formation, particularly reflecting the transition from cortical to cancellous bone. Micro-computed tomography and histological analyses have demonstrated that gradient scaffolds not only facilitate robust new bone growth but also increase the expression of osteogenic markers such as osteocalcin and alkaline phosphatase, indicating active bone formation and mineralization. Moreover, these scaffolds enhance angiogenesis, an essential factor for successful bone regeneration, by boosting the expression of vascular endothelial growth factor (VEGF) and other endothelial markers [192].

#### 3.1.6. Poly(Glycolic Acid) (PGA)

PGA is a synthetic, biodegradable polymer that has shown great potential in the field of bone regeneration. Its high crystallinity and mechanical strength make it a suitable candidate for fabricating scaffolds that support BTE. PGA’s Young’s modulus of 7.0 GPa and melting temperature of approximately 230 °C contribute to its robustness compared to other biodegradable polymers, enabling it to better mimic the mechanical properties of human bones [25,197].

Recent advancements have focused on the development of 3D-printed PGA scaffolds combined with HAp NPs to enhance bone regeneration. HAp, a naturally occurring mineral form of calcium apatite, is known for its excellent osteoconductivity, biocompatibility, and ability to bond directly to bone [26]. However, sintered HAp alone suffers from poor formability and mechanical properties. Integrating HAp with PGA addresses these limitations, creating a composite material that benefits from the structural integrity of PGA and the osteoconductive properties of HAp [25].

Taegyun Yeo et al. (2020) reported that PGA/HAp composite scaffolds are designed to degrade in a controlled manner, synchronizing with the rate of new bone tissue formation. This ensures that as the scaffold material degrades, it is replaced by new bone, maintaining the structural integrity of the regenerating tissue. The degradation process of PGA involves two stages: initial hydrolysis of the amorphous regions, followed by breakdown of the crystalline regions, allowing for gradual weight loss and scaffold resorption over time [25].

In vitro studies have confirmed the enhanced performance of PGA/HAp scaffolds in supporting the attachment, proliferation, and differentiation of osteoblasts. These cells are crucial for bone formation, producing and depositing the ECM necessary for bone regeneration. Enhanced calcification has been observed in PGA/HAp scaffolds with higher HAp content, particularly those containing 12.5 wt% and 15 wt% HAp, indicating a favorable environment for BTE [25].

Overall, the integration of PGA with HAp NPs in 3D-printed scaffolds presents a promising strategy for bone regeneration. This composite approach not only improves the mechanical properties and biocompatibility of the scaffolds but also supports the natural bone healing process, offering a feasible solution for patient-specific bone regeneration in clinical applications [25].

### 3.2. Chitosan NPs for Bone Regeneration

Chitosan, a natural polysaccharide derived from chitin, has been extensively studied for its potential applications in TE and bone regeneration. Due to its biodegradability, biocompatibility, and ease of modification, chitosan has emerged as a promising candidate for developing degradable biocompatible guided bone regeneration membranes [197].

Ankit J. et al. (2014) demonstrated the potential of chitosan nanofiber membranes, both with and without genipin crosslinking, as compared to commercial collagen membranes. Their findings showed no statistically significant difference in tissue reaction between chitosan and collagen membranes. Minimal inflammation was observed in 57–74% of the membranes across all groups. Notably, most chitosan membranes persisted for 16–20 weeks, while most collagen membranes were resorbed by 12–16 weeks, indicating that chitosan membranes degrade more slowly than collagen membranes. The overall tissue response to chitosan nanofiber membranes, with and without genipin crosslinking, was similar to that of the control commercial collagen membrane, highlighting chitosan’s potential as a guided bone regeneration material [197].

Chitosan NPs, particularly when incorporated into asymmetric collagen-chitosan membranes, are highly effective for bone regeneration. These NPs are ideal for drug delivery applications due to their biocompatibility and biodegradability [198]. The addition of aspirin-loaded chitosan NPs to these membranes allows for sustained drug release, enhancing bone regeneration by exploiting the osteogenic properties of aspirin [176]. The membranes consist of a dense chitosan layer and a loose collagen layer, designed to optimize cell adhesion and proliferation, while simultaneously preventing the ingrowth of soft tissue into the bone defect area. This configuration facilitates the proliferation and osteogenic differentiation of BMSCs more effectively than other materials. In vivo tests on rat cranial defect models have shown that these membranes significantly enhance bone regeneration, underscoring the potential of chitosan NPs in advanced bone repair therapies [126].

Additionally, chitosan NPs have been explored in dental applications, particularly for their antimicrobial and remineralizing properties. A study by Wu L. et al. (2018) investigated the feasibility of using amorphous calcium phosphate (ACP) formed in situ from chitosan calcium microspheres and phosphate ions in water during brushing for caries control. Their findings indicated that the Chi-ACP paste significantly enhanced remineralization and provided antimicrobial benefits, underscoring the potential of chitosan-based NPs in BTE applications as well [108].

## 4. Conclusions

NPs represent a significant advancement in BTE, offering promising solutions for overcoming the limitations of traditional bone grafts and scaffolds. This study highlights the versatile applications of NPs, including polymers, metals, ceramics, and composites, in enhancing osteogenesis and angiogenesis by mimicking the ECM nanostructure. The integration of NPs such as reduced graphene oxide, HAp, and carbon nanotubes into scaffolds has demonstrated significant improvements in mechanical properties, cell proliferation, and differentiation. Despite the promising results, challenges related to cytotoxicity, biodistribution, and immune responses remain. Addressing these issues through surface modifications and functionalization with biocompatible molecules can enhance the biocompatibility and efficacy of these nanomaterials. Future research should focus on long-term in vivo studies in animal models to evaluate the safety and efficacy of nanoparticle-based scaffolds, as well as exploring synergistic effects with other bioactive molecules or growth factors to further enhance bone regeneration therapies.

Despite the advancements in nanoparticle-based BTE, several challenges persist. The primary concerns include cytotoxicity, biodistribution, and immune responses associated with various NPs. Addressing these issues requires comprehensive surface modifications and functionalization with biocompatible molecules to enhance their safety profile. Additionally, the long-term effects of these NPs in vivo in animal models remain to be thoroughly investigated. Future research should prioritize extended in vivo studies in animal models to evaluate the safety and efficacy of these materials over time. Moreover, exploring the synergistic effects of combining NPs with other bioactive molecules or growth factors could further amplify their osteogenic potential. This includes investigating the integration of NPs with advanced drug delivery systems to provide targeted and sustained release of therapeutic agents. The development of multifunctional scaffolds that can support both osteogenesis and angiogenesis is also a critical area for future research.

## Figures and Tables

**Table 2:** This table categorizes different shapes available for gold NPs, silver NPs, aluminum NPs and nanosilica.

	Nanoparticle Shapes										
Gold Np	Nanocluster 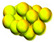	Nanoshell 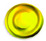	Nanobranch 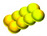	Nanotriangle 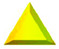	Nanocube 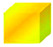	Nanohexagon 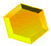	Nanopentagon 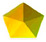	Nanorods 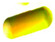	Nanosphere 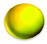	Nanocage 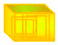	Nanostars 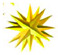
Silver NP	Nanospheres 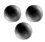	Nanoshell 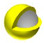	Nanorice 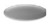	Nanotriangle 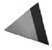	Nanocube 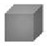	Truncated Octahedron 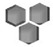	Nanostar 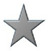	Nanorods 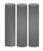	Nanodisk 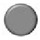	Nanowires 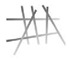	
Au NPs	Nanospheres 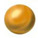	Nanoshell 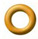		Nanoflower 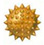		Nanorod 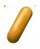		Nanocube 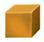		Nanocage 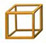	
Nano silica	Capsule		Rice		Cube				Rhombus		
	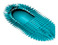		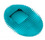		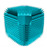				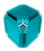		
